# Toward a better judgment of item relevance in progress testing

**DOI:** 10.1186/s12909-017-0989-x

**Published:** 2017-09-05

**Authors:** Xandra M. C. Janssen-Brandt, Arno M. M. Muijtjens, Dominique M. A. Sluijsmans

**Affiliations:** 1 Faculty of Health, Zuyd University of Applied Sciences, Nieuw Eyckholt 300, 6419 DJ Heerlen, The Netherlands; 2 Department of Educational Development and Research, Maastricht University, Universiteitssingel 60, 6229 ER Maastricht, The Netherlands

**Keywords:** Item relevance, Midwifery education, Progress testing, Rubric

## Abstract

**Background:**

Items must be relevant to ensure item quality and test validity. Since “item relevance” has not been operationalized yet, we developed a rubric to define it. This study explores the influence of this rubric on the assessment of item relevance and on inter-rater agreement.

**Methods:**

Members of the item review committee (RC) and students, teachers, and alumni (STA) reassessed the relevance of 50 previously used progress test (PT) items and decided about their inclusion using a 5-criteria rubric. Data were analyzed at item level using paired samples t-tests, Intraclass Correlation Coefficients (ICC), and linear regression analysis, and at rater level in a generalizability analysis per group.

**Results:**

The proportion of items that the RC judged relevant enough to be included decreased substantially from 1.00 to 0.72 (*p* < 0.001). Agreement between the RC and STA was high, with an ICC of >0.7 across items. The relation between inclusion and relevance was strong (correlation = 0.89, *p* < 0.001), and did not differ between RC and STA. To achieve an acceptable inter-rater reliability for relevance and inclusion, 6 members must serve on the RC.

**Conclusions:**

Use of the rubric results in a stricter evaluation of items’ appropriateness for inclusion in the PT and facilitates agreement between the RC and other stakeholders. Hence, it may help increase the acceptability and validity of the PT.

## Background

One of the criteria for ensuring the quality of test items is that items must be relevant [1]. This means that they should adequately reflect specific content that is consistent with predefined learning goals and professional practice [2]. Ensuring item relevance is important for several reasons. First, it is a necessary precondition for ensuring the validity of a test as a whole. Messick has broadly defined validity as *“*an integrated evaluative judgment of the degree to which empirical evidence and theoretical rationales support the adequacy and appropriateness of inferences and actions based on test scores or other modes of assessment” [3]. Although validity is currently being revisited, it is still common practice to consider content, criterion, and construct validity as its integral parts [4, 5]. “Content validity” refers to the extent to which the content of test items reflects the intended content domain, that is, the content which has been taught [4–7]. Consequently, content validity and item relevance are inextricably linked: content validity will be seriously compromised when a test includes irrelevant items [8–10]. Second, reduced relevance of test content to professional practice casts doubts on the validity of inferences about licensing and certification [11, 12]. Moreover, when too many test items of doubtful relevance are included, inferences drawn from students’ scores will be less accurate and defensible for professional practice, thereby decreasing the predictive validity of the test [4, 13]. A third argument is that the presence of irrelevant items may point to a disagreement between assessment and educational objectives, thereby creating a “hidden curriculum” which deviates from the educational objectives [14, 15]. In order to prevent tests from assessing a “hidden curriculum,” one must have a clear definition of what exactly constitutes a “relevant item.”

What complicates matters, however, is that item relevance is strongly dictated by item writers’ individual strategies based on own practice experience [11]. To increase the validity of tests, educational institutes often commission a review committee consisting of independent experts in various content areas of the curriculum to write, discuss, and review the items [6, 7, 10, 16]. In deciding which items to include, this review committee uses a blueprint that specifies how many of the test questions should pertain to each subdomain. Use of such a blueprint, however, does not preclude disagreement between reviewers about the relevance of items. Item rating procedures, such as Likert-type scales to rate the extent to which items reflect educational objectives, or matching tasks to match items with one of the test objectives may offer some relief [6]. General guidelines about how to validate the evidence for item content, by using a blueprint, for example, may also be instrumental [7]. Yet, these are not a panacea as they fail to provide exact details or criteria for achieving more clarity and consistency among raters regarding item relevance [6]. Since “item relevance” has not been operationalized yet, it is difficult for item writers and reviewers to fully grasp its meaning [17].

Item relevance is especially important for progress testing, a longitudinal examination reflecting the cognitive exit qualifications of the curriculum. Maastricht University and the University of Missouri–Kansas City were the first to introduce the progress test (PT) in the late 1970s and many university programs in the medical and health sciences have since followed suit [10, 15, 17–22]. PT items may apply to the entire content of the educational program, thereby strongly discouraging superficial rote learning [23, 24]. Since the test targets long-term and functional knowledge, only a deep approach to learning will pay off [8, 20, 23, 24]. The broad nature of exit qualifications, however, makes it inherently difficult to evaluate the relevance of items for progress testing [10, 15].

The lack of transparent criteria for determining item relevance often leads to the incorporation of trivial, detailed and specialized knowledge items in the PT. These types of items do little to enhance learning and bear no significant relation to a specific profession. Instead, they test the memorization of isolated facts, which is beyond the scope of the PT. By assessing the exit qualifications of the curriculum, the PT in effect aims to test the ability to manage complex problems, the capacity to apply knowledge to novel problems and the understanding of essential concepts [9, 10, 13, 15, 21]. Trivial items are often perceived as difficult, and, consequently, their excessive use increases the inter-test variation in student performance, which is already elevated due to the lack of test equating [10, 14, 15, 19]. Likewise, chances of students and staff accepting the test as an instrument that measures the program’s broad exit qualifications decrease with the complexity of writing items that are not directly related to a particular course. The various PT stakeholders (item writers, reviewers, students) hold widely differing views on what they regard as relevant for certification [10].

Hence, reaching a consensus agreement on item relevance for progress testing is challenging [9, 10, 17]. In the absence of tools to define item relevance, in this study we decided to explore the use of an existing rubric which might aid the process of defining item relevance. “Rubrics” are descriptions of discrete scoring levels produced to guide the qualitative assessment of performance [25–28]. A potential benefit of using a rubric is increased consistency of ratings across the assessed aspects as well as between raters [25, 26].

Developed at Maastricht University [2011 letter from L.W.T. Schuwirth to the review committee of the medical PT; unreferenced personal communication], the rubric we used in this study includes five relevance criteria which were defined by consensual expert agreement to encourage a more consistent and accurate interpretation of item relevance [10]. It was specifically designed to offer a more comprehensive definition of “item relevance”, leading to more sound decisions about inclusion of items in a test, while increasing agreement on item relevance between stakeholders.

The purpose of this study is to explore the extent to which the rubric can help improve the item review process by reducing the number of irrelevant items included in a test and by facilitating agreement on item relevance and inclusion between reviewers, staff, and students, both at school and in the work field. To this end, the paper addresses the following research questions:

1. Does use of the relevance rubric as a supplement to the blueprint influence the decision to include an item in the PT?
2. Does use of this rubric lead to agreement between groups (review committee vs. students, teachers, and alumni) on item relevance and inclusion of items in the PT?
3. How are item relevance and inclusion of items in the PT related in the aforementioned two groups?
4. What is the level of inter-rater agreement on item relevance and inclusion *within* these two groups?
5. How many raters should serve on the review committee in order to achieve a reliable assessment of item relevance and reach a sensible decision on whether or not to include an item in the PT?

## Methods

### Design and setting of the study

We conducted this study at the Faculty of Health, department Midwifery Education & Studies (DMES) at Zuyd University of Applied Sciences in Maastricht, the Netherlands. The DMES has used Problem-Based Learning and a PT since the early 1990s. We chose this context because the faculty’s students and staff frequently complain about the relevance of items in the progress test. The level of difficulty also differs greatly between tests, making it hard to pin down students’ individual progress. At the DMES a progress test review committee (RC) judges the relevance of items in a PT and decides about their inclusion in the test. The committee is composed of a chair who is responsible for item and test construction, currently a medical doctor who is also the main researcher of this study, and three midwives who decide on test content. The construction of the PT is highly standardized: it is based on a blueprint and the RC meets regularly to discuss all items before including them in the PT.

The PT is administered four times per year to second-, third-, and fourth-year students for summative purposes. It contains 150 true/false items with the response options “yes,” “no,” and “I do not know the answer.” The results are obtained on the basis of formula scoring: a correct answer will give a score of 1, an incorrect answer a score of −1, while “abstention” leads to a score of 0. This scoring method intends to reduce the occurrence of wild guessing and the resulting noise that obfuscates measurement [10, 19].

### Participants

Participants were various stakeholders involved in the PT: fourth-year students, teachers, alumni, and the RC. We selected fourth-year students to judge the items in terms of their relevance to the curriculum because of their familiarity with most parts of the curriculum content. We specifically invited teachers with a midwifery background, because they know which competences a midwife should have and the content of the curriculum. Alumni were included because of their familiarity with the PT and their ability to review the test items in light of their experience as a midwife. And, finally, we included the members of the RC who all had a background in midwifery, because of their experience in defining item relevance for the PT without using the rubric.

We invited 30 students (27 of which were randomly selected by the computer from a list of 52 fourth-year students from the 2012–2013 academic year, three others voluntarily offered to participate), all teachers of the department with a midwifery background (*n* = 19), 40 alumni (randomly selected by the computer from a population of 87 graduates between 2010 and 2012), and all members of the RC with a midwifery background (*n* = 3) to participate. Twenty-one students (64%), 15 teachers (79%), nine alumni (23%) and all three members of the RC (100%) participated as the main raters involved in the PT. With a total of 48 participants, the response rate amounted to 51%.

### Materials

#### PT items

We randomly sampled 50 PT items from a progress test which had recently been administered to explore how use of the rubric would affect the assessment of item relevance and item inclusion. As part of an official PT, the 50 items had already been assessed by the RC using the blueprint but not the rubric, and were found sufficiently relevant to be included in the PT.

#### Rubric to assess item relevance

The rubric we used to assess item relevance was developed at Maastricht University in accordance with guidelines for rubric construction [26–28] and has been in unofficial use since 2011. It contains five criteria for item relevance, with three levels of item relevance specified for each criterion: the item can be rated as “not relevant,” “somewhat relevant,” or “very relevant” to the criterion listed. The five criteria for relevance are: 1) medical knowledge: a thorough study and understanding of medical knowledge is required to answer the item correctly, 2) ready knowledge: the knowledge is needed at any moment, 3) incidence in practice: the knowledge is needed in many situations, 4) prevalence or high risk: the knowledge is needed to manage high-prevalence or high-risk situations, and 5) knowledge foundation in the medical curriculum: the knowledge forms the basis of one or more medical concepts. Table 1 presents the rubric for item relevance as adapted to the context of midwifery education.

**Table 1:** Rubric to assess item relevance (adapted from Schuwirth LWT 2011, by Janssen-Brandt XMC, 2012)

Criterion	Not relevant	Somewhat relevant	Very relevant
Midwifery knowledge	General knowledge a layperson would know.	Specific to midwifery but also known to an interested layperson.	Requires a thorough study and understanding of the field of midwifery.
Ready knowledge	Not necessary to be remembered and can be looked up quickly.	Can be looked up quickly, but graduates should know it.	Needed at the ready at any moment of the day.
Incidence in practice	Not important for any midwifery (not just clinical) situation.	Only needed in rare or extreme practical midwifery situations.	Needed in many practical midwifery situations.
Prevalence or high risk	Found very rarely in midwifery practice or is low risk.	Not essential for managing high prevalence or high-risk midwifery situations.	Essential for managing high-prevalence or high-risk midwifery situations.
Knowledge foundations in the Midwifery curriculum	An isolated fact not needed to understand other concepts in the curriculum.	Necessary to understand other concepts, but can be forgotten.^a^	Forms the basis of one or more concepts and has to remain as explicit knowledge.^b^

^a^E.g., Bohr/Haldane effect to understand why hemoglobin dissociates from O_2_ in the tissues and takes up O_2_ in the lungs

^b^E.g., the Frank-Starling mechanism in the context of congestive heart failure, or for midwifery the labor mechanism in the context of abnormal position or presentation of the presenting part

#### Variables

We invited participants to assess the relevance of each of the 50 items using the rubric and then answer the following two questions: “What is the overall relevance of this item?” (Item relevance variable: not relevant = 0, somewhat relevant = 0.5, very relevant = 1), and “Should the item be included in the PT?” (Item inclusion variable: no = 0, yes = 1).

### Procedure

We sent students an information email with an invitation to participate and an informed consent form. Those who agreed to participate attended a training session about the use of the rubric, which was followed by a two-hour session in which they independently reviewed and rated each of the 50 items in terms of their relevance and fitness for inclusion using the rubric. Teachers and members of the RC were informed and trained in a team meeting, during which we explained to them how to use the rubric. Afterwards, we sent them an invitation by email, along with an informed consent form, the sample of 50 PT items, rubric, and scoring form. We instructed this group to complete the scoring form independently and to return it to the researcher within three weeks. Finally, the alumni were also invited and asked for their informed consent by email. The main researcher sent them written instructions on how to use the rubric, along with the 50 progress test items, rubric, and scoring sheet. They were kindly requested to complete the form independently and to return it to the researcher within four weeks.

### Data analysis

To answer the first three research questions, we analyzed the data at the item level. For each of the 50 items we computed the average item relevance per group, that is, the review committee (RC) on the one hand and students, teachers, and alumni (STA) on the other. The resulting variables are referred to as “RC item relevance,” and “STA item relevance,” respectively. Similarly, we obtained the average ratings for item inclusion per item for each of the two groups, resulting in the variables “RC item inclusion” and “STA item inclusion,” respectively. In order to answer research question 1, we tested the difference between 1 (representing the earlier decision of the RC to include the items) and the recently computed RC item inclusion in a one-sample t-test. A *p*-value of 0.05 or smaller was considered to indicate a significant result, implying that the judgment of the review committee in the new procedure using the rubric had changed compared to the earlier judgment. Since the null hypothesis in this test was “population mean Inclusion = 1,” we were testing against the unidirectional alternative hypothesis “population mean Inclusion <1”, and therefore used a one-sided t-test.

In order to answer research question 2 we performed a two-phase analysis of the inter-group differences in ratings of item relevance. First, we applied a paired samples t-test to the 50 paired ratings (RC item relevance−STA item relevance), quantifying the mean between-group difference in ratings of item relevance and inclusion. We wished to investigate whether STA still had lower ratings on relevance and inclusion than the RC after the intervention. Consequently, the alternative hypothesis for item relevance read “population mean STA item relevance - population mean RC relevance < 0,” which formula was the same for inclusion, and we therefore used one-sided t-tests. Second, we calculated the Intraclass Correlation Coefficient (ICC) for the set of 50 paired ratings (RC item relevance−STA item relevance) to get an indication of the level of intergroup agreement on item relevance. The ICC compares the variance between items, called the “variance of interest,” with the sum of the variance of interest and the variance within items (representing the between-group disagreement), so an ICC value near 1 indicates a high level of agreement, i.e., a high intergroup reliability of the ratings. In general, a reliability of 0.70 is considered sufficient, whereas a reliability of 0.80 or higher is recommended for high-stakes decisions [29]. We performed similar analyses to make inferences about item inclusion.

In order to answer research question 3 we performed an analysis on item level, item inclusion being the dependent variable and item relevance and group (0: STA, 1: RC) the independent variables. Note that on item level the value of both relevance and inclusion is obtained by calculating the mean of several raters, and can therefore be treated as a continuous variable in either case. As a result, we performed a linear regression analysis to investigate the relationship. In this analysis we included not only group and item relevance, but also the interaction of group and relevance as independent variables. This allowed us to investigate whether the relationship between inclusion and relevance differed across the two groups.

In order to answer research questions 4 and 5, we performed a generalizability analysis of the data for the item relevance and item inclusion variables at the individual rater level for each of the two groups [30]. In doing so, we used an items-crossed-with-raters design, obtaining estimates of the variance components *V* (*item*), *V* (*rater*), and *V* (*item*, *rater*); these represent the variance of interest, the general between-rater disagreement, and the item-specific between-rater disagreement, respectively. As we were interested in the absolute generalizability of item ratings, we calculated the *Phi* coefficient, which is defined as

$$ Phi=\frac{V(item)}{V(item)+V(rater)/N+V\left( item, rater\right)/N} $$

where *N* is the number of raters. One can calculate *Phi* for the actual number of raters to know the absolute inter-rater reliability in the real situation. Additionally, one may calculate *N* for any given value of *Phi*, thus revealing how many raters are required to reach a certain level of reliability. This is called a decision analysis, which tells us how many RC members are required to obtain a reliability of 0.70, thereby providing an answer to research question 5. To analyze the data, we used statistical package SPSS, version 22.

## Results

Table 2 sets out the mean of all 50 item ratings in terms of relevance (1st row) and inclusion (2nd row) and their standard deviations for each of the two groups (RC and STA). We found both groups to present similar mean ratings, not only of item relevance (RC: 0.610 vs. STA: 0.626), but also of item inclusion (RC: 0.720 vs. STA: 0.729), and, consequently, differences between groups were not significant (Relevance: −0.016, *p* = 0.26; Inclusion −0.009, *p* = 0.37). The mean rating of item inclusion appeared to be significantly lower than 1 for both groups. In the case of the RC this finding is particularly striking, since it was by their own agency that all 50 items had previously been included in the PT, and hence, at the time their ratings of item inclusion equaled 1. As a corollary, it transpires that the RC evaluates the appropriateness (in relation to relevance) of items for inclusion in the PT more strictly when using the rubric compared to the earlier procedure, suggesting that research question 1 should be answered in the affirmative.

**Table 2:** Average ratings of items by RC on the one hand, and STA on the other

Variable	RC N raters = 3			STA N raters = 45			RC/STA difference	
	M	SD	*p* ^1)^	M	SD	*p* ^1)^	Mrc-Msta	*p* ^2)^
Relevance	0.610	0.304		0.626	0.264		−0.016	0.26
Inclusion	0.720	0.325	0.0003	0.729	0.281	0.0003	−0.009	0.37

^1)^One-sided *p*-value of a one-sample t-test against the null hypothesis: population mean rating of inclusion = 1, which corresponds to the before-study judgment by the RC on each of the 50 items in the sample

^2)^One-sided *p*-value of a paired samples t-test against the null hypothesis: mean RC/STA difference = 0

Table 3 presents the intergroup agreement on the rating of 50 PT items in terms of their relevance and inclusion, as measured by the Intraclass Correlation Coefficient (ICC). With ICCs of 0.82 and 0.81 for relevance and inclusion, respectively, agreement between the RC and STA on relevance and inclusion appeared very high, and, with that, research question 2 has been answered. The variance component of the group, V(group), was found to be close to 0, indicating that there was hardly any systematic between-group difference in the assessment of item relevance and item inclusion. This result corresponds with the non-significant between-group differences found for the paired samples t-tests presented in Table 2.

**Table 3:** Intergroup agreement by the RC on the one hand, and the STA on the other

Variable	Variance Components			Intraclass Correlation Coefficient (ICC)
	V(item)	V(group)	V(item, group)	
Relevance	0.066	0.000	0.015	0.82
Inclusion	0.074	0.000	0.018	0.81

The regression analysis investigating the relationship between inclusion and relevance at item level (research question 3) did not reveal any significant contributions of group, nor did it yield any significant interaction between group and relevance. This implies that the relationship between inclusion and relevance did not differ across the RC and STA. The regression coefficient for relevance amounted to 0.95 and was highly significant (t-value 19.2, *p* < 0.001), the correlation coefficient accounting for 0.89 hinting at a strong relationship.

The results that reveal the level of inter-rater agreement on item relevance and item inclusion *within* the RC and STA, providing an answer to research question 4, are presented in Table 4. The columns “V(item),” “V(rater),” and “V(item, rater)” present the variance components obtained in the generalizability analysis. When we compare these *intra*group data with the *inter*group components of Table 3, then, not surprisingly, we find the variances of interest or “V(item)” to be practically identical, while both of the “error” variance terms are larger for the rater level results in Table 4. It is plausible that part of the rater level error was “averaged out” by the aggregation to the group level. As a consequence of the elevated error terms at rater level, the assessment of relevance and inclusion can only be sufficiently reliable if the number of raters is increased. Upon closer inspection of Table 4, we find similar inter-rater reliabilities (*Phi* coefficients) for item relevance in both groups (RC and STA), regardless of the number of raters. Reliabilities for item inclusion, by contrast, result much higher in the STA group than in the RC, suggesting that the members of the RC disagree more on item inclusion than do the STA raters.

**Table 4:** Inter-rater agreement (Phi coefficient) on the rating of PT items within each group of raters

Group	Variable	Variance Components			Phi coefficient for varying numbers of raters					
		V(item)	V(rater)	V(item,rater)	3	4	5	6	7	10
RC	Relevance	0.068	0.005	0.073	0.72	0.78	0.81	0.84	0.86	0.90
	Inclusion	0.063	0.017	0.130	0.56	0.63	0.68	0.72	0.75	0.81
STA	Relevance	0.068	0.008	0.075	0.71	0.77	0.80	0.83	0.85	0.89
	Inclusion	0.077	0.009	0.114	0.65	0.71	0.76	0.79	0.81	0.86

To answer research question 5, we merely need to read Table 4 backward, by first looking up a *Phi* coefficient that meets or exceeds the required level, and then locating the corresponding number of raters needed to reach that level in the upper row. The *Phi* coefficients listed in Table 4 demonstrate that, for the assessment of relevance, three RC members are needed to reach a level of 0.70. For the inclusion judgment a reliability of 0.7 would require at least six members. For the STA group, results for relevance are similar to those for the RC group: three raters would yield a reliability level of 0.70. For inclusion, however, four STA members would suffice to reach this level of reliability.

## Discussion

The purpose of this study was to explore the extent to which the rubric can help improve the item review process by reducing the number of irrelevant items included in a test, and by facilitating agreement on item relevance and inclusion between reviewers, staff, and students, both at school and in the work field. Results reveal that the use of a rubric to define item relevance in progress testing indeed alters the judgment on inclusion. The RC had previously decided to include all of the 50 items used in this study in a PT, so, back then, these items had a mean inclusion score of 1. It has been demonstrated that the number of items included in a test decreases dramatically with use of the rubric. Seemingly, the RC evaluates the appropriateness (in relation to relevance) of items for inclusion in the PT more strictly when using the rubric. As a result, fewer irrelevant items are included in the progress test, improving the validity of the test [9, 10, 15, 21].

We also found the RC and STA to agree strongly on relevance and inclusion of the 50 PT items when using the rubric, as reflected in their average ratings. Another similarity that the two groups shared was that they had comparable relationships between relevance and inclusion. Systematic between-group differences in the assessment of item relevance and item inclusion were largely absent, suggesting that both groups were almost equally strict on the rating of item relevance. Since “item relevance” has not been operationalized yet, it is difficult to reach agreement on its definition [17]; the rubric clearly represents an important step in its operationalization and, accordingly, helps improve its assessment.

The groups agreed more strongly on item relevance than on item inclusion. To obtain reliable results, the assessment of item inclusion therefore required more raters than that of item relevance. Since the inter-rater agreement on item inclusion was higher within the STA group compared to the RC, the latter needed more raters than did the STA to come to a reliable decision about item inclusion. This indicates that the minimum relevance level required for inclusion (the norm for inclusion) varied more widely within the RC than in the STA group, necessitating at least six RC members for reliable decisions on item inclusion.

Although the results of this study are promising, some limitations need to be considered. First, the re-evaluation of used and approved PT items using the rubric may have restricted the range of relevance. Items of little relevance were not included in the sample because they had been previously rejected by the RC. However, students still complained about a lack of relevance which suggests that the PT contained enough items that were irrelevant, mitigating the weight of this limitation. Second, the small size of the RC may have complicated the interpretation of the results, a difficulty we could not overcome as this was the actual current size of the RC. Despite this, the results concerning agreement between the RC and STA when using the rubric are compelling since the various aspects under scrutiny all consistently point to strong agreement. Third, we employed different methods to instruct stakeholders on how to use the rubric and rate the items: while RC, students, and teachers received the instructions during a meeting, alumni did so by email. Moreover, we only supervised students during item evaluation, as we expected the other stakeholders to be capable of rating the items independently as per the instructions received. This may have slightly influenced the results, as students may have changed their minds about the relevance of items had they been given the opportunity to discuss with their peers. Fourth, the use of true/false items may have affected the relevance rating process as literature shows that such dichotomous items fairly often test trivial factual knowledge and discourage deep learning about the subject [31]. It would therefore be interesting to replicate the study for a PT with multiple-choice items. Currently, there is a trend toward using single-best-answer multiple-choice questions with the PT at DMES, thus offering an excellent opportunity for a follow-up study with MCQs.

The findings of the present study provide several additional opportunities for further research. It may be interesting, for instance, to investigate if students appreciate the PT better when its items have been included based on the rubric. Second, the size of the RC was very small, calling for additional studies to assess the reliability of the rubric. Third, it has been demonstrated that there is variation in the relevance-to-inclusion ratio. These variables may be found to correlate in a way that differs from rater to rater. More specifically, when deciding whether or not to include an item in the PT, raters may be attaching different weights to the various relevance criteria. This could lead one reviewer to include an item based on a high score on one criterion, and another to exclude that same item on the basis of a similar score, but different interpretations of its value. Further research is needed to understand this. Finally, future studies should investigate whether the results can be extrapolated to a PT in medical education. Midwives differ from medical teachers in that they are more or less experts in the entire midwifery domain, while doctors are generally experts in a certain subdomain. Basic scientists, for example, are known to have more difficulty in defining core knowledge required for graduation [9]. As a result of their familiarity with the entire midwifery curriculum, midwives might agree more on item relevance than their medical counterparts with expertise on subdomains. This could lead to a higher level of agreement among midwives compared to other experts.

This study underlines that the literature on item relevance is inconclusive. Consequently, item writers and reviewers do not have adequate tools that allow for a systematic approach to defining item relevance. This complicates test construction, especially of a progress test which is based on curriculum objectives [10]. As this study has demonstrated, the rubric to assess item relevance can serve as a framework for all midwifery schools using a progress test and possibly also for other types of (health) schools, e.g., medical schools, especially when there is discussion about item relevance. Item writing guidelines and an interdisciplinary review process have been shown to improve item quality by offering suggestions to strengthen the item [16]; the use of the rubric, by contrast, contributes to the discussion of item relevance with further improvement of item and test quality. Further research will need to focus on the validity and feasibility of the rubric for appraising item relevance, on the extrapolation of results to other medical education practices, and on stakeholders’ acceptance of the PT with rubric use (that is, more satisfaction among students and less complaints about item relevance).

## Conclusion

Use of a rubric reduces the number of irrelevant items included in the PT and facilitates agreement between stakeholders on the relevance and inclusion of items in progress testing. For a reliable decision on whether or not an item is sufficiently relevant to be included in a (midwifery) progress test the review committee should consist of at least 6 members. The inclusion of more relevant items may help to further increase the validity and acceptability of the PT.

## Abbreviations

DMES: Department Midwifery Education & Studies
ICC: Intraclass Correlation Coefficients
PT: Progress test
RC: Review committee
STA: Students, teachers, alumni
V: Variance component
